# Supplementary material for: Time-varying SUVr reflects the dynamics of dopamine increases during methylphenidate challenges in humans
Source: Commun Biol. 2023 Feb 10;6:166. doi: 10.1038/s42003-023-04545-3 (PMC9918528; doi:10.1038/s42003-023-04545-3)
Supplement: Supplementary file 2 — Reporting Summary [file 42003_2023_4545_MOESM2_ESM.pdf]

nature research

Corresponding author(s): Dardo Tomasi

Last updated by author(s): 1/24/2023

Reporting Summary

Nature Research wishes to improve the reproducibility of the work that we publish. This form provides structure for consistency and transparency in reporting. For further information on Nature Research policies, see our Editorial Policies and the Editorial Policy Checklist.

Statistics

For all statistical analyses, confirm that the following items are present in the figure legend, table legend, main text, or Methods section.

n/a	Confirmed	
☐	☒	The exact sample size (n) for each experimental group/condition, given as a discrete number and unit of measurement
☐	☒	A statement on whether measurements were taken from distinct samples or whether the same sample was measured repeatedly
☐	☒	The statistical test(s) used AND whether they are one- or two-sided<br>*Only common tests should be described solely by name; describe more complex techniques in the Methods section.*
☐	☒	A description of all covariates tested
☐	☒	A description of any assumptions or corrections, such as tests of normality and adjustment for multiple comparisons
☐	☒	A full description of the statistical parameters including central tendency (e.g. means) or other basic estimates (e.g. regression coefficient) AND variation (e.g. standard deviation) or associated estimates of uncertainty (e.g. confidence intervals)
☐	☒	For null hypothesis testing, the test statistic (e.g. F, t, r) with confidence intervals, effect sizes, degrees of freedom and P value noted<br>*Give P values as exact values whenever suitable.*
☒	☐	For Bayesian analysis, information on the choice of priors and Markov chain Monte Carlo settings
☒	☐	For hierarchical and complex designs, identification of the appropriate level for tests and full reporting of outcomes
☒	☐	Estimates of effect sizes (e.g. Cohen's d, Pearson's r), indicating how they were calculated

Our web collection on statistics for biologists contains articles on many of the points above.

Software and code

Policy information about availability of computer code

Data collection	PET and MRI data were collected using Siemens Biograph mMR software version VE11P; High rating button press responses were collected using E-Prime Version 3.0.
Data analysis	Imaging data was analyzed using FSL (FMRIB, Oxford), Freesurfer (MGH), and SPM (UCL). Simulations and curve fitting were carried in IDL (L3HARRIS). Statistical analyses were carried in R using Rstudio. G*power was used for statistical power calculations.

For manuscripts utilizing custom algorithms or software that are central to the research but not yet described in published literature, software must be made available to editors and reviewers. We strongly encourage code deposition in a community repository (e.g. GitHub). See the Nature Research guidelines for submitting code & software for further information.

Data

Policy information about availability of data

All manuscripts must include a data availability statement. This statement should provide the following information, where applicable:

- Accession codes, unique identifiers, or web links for publicly available datasets
- A list of figures that have associated raw data
- A description of any restrictions on data availability

Dynamic DA release and peak 'high' rating scores will be made available in Figshare before publication.

Field-specific reporting

Please select the one below that is the best fit for your research. If you are not sure, read the appropriate sections before making your selection.

☒ Life sciences ☐ Behavioural & social sciences ☐ Ecological, evolutionary & environmental sciences

For a reference copy of the document with all sections, see nature.com/documents/nr-reporting-summary-flat.pdf

Life sciences study design

All studies must disclose on these points even when the disclosure is negative.

Sample size	Within-subjects: Assuming alpha = 0.05 (threshold probability for rejecting the null hypothesis; i.e. Type I error rate) and beta = 0.2 (probability of failing to reject the null hypothesis under the alternative hypothesis; i.e. Type II error rate), 48 time windows (number of within-subjects measures) allowed us to reject departures from the model (Eqn [1]) of effect size w>0.8, using chi-square goodness-of-fit test). Across-subjects: with 20 participants, alpha = 0.05 and beta = 0.2, we were able to detect correlations between dopamine time-to-peak and high ratings with |R|>0.53, and differences in dopamine increases between oral- and iv-methylphenidate conditions with effect size>0.13, using within-subjects ANOVA with 48 time windows.
Data exclusions	The analysis of interindividual variability was restricted to the putamen because model fitting to individual delta SUVr time courses was deemed unreliable in other striatal regions, particularly for oral-methylphenidate that demonstrated lack of fit convergence in 16 (caudate) or 14 (ventral striatum) individuals, due to the lower signal-to-noise and smaller size of these regions.
Replication	The model (Eq [1]) used to assess dopamine increases with positron emission tomography in humans, also reproduced dopamine increases measured with microdialysis in rats and monkeys which were published by others.
Randomization	The session order was randomized across participants.
Blinding	Participants and researchers were blind to the nature of the stimulant drug (methylphenidate/placebo).

Reporting for specific materials, systems and methods

We require information from authors about some types of materials, experimental systems and methods used in many studies. Here, indicate whether each material, system or method listed is relevant to your study. If you are not sure if a list item applies to your research, read the appropriate section before selecting a response.

Materials & experimental systems

n/a	Involved in the study	
☒	☐	Antibodies
☒	☐	Eukaryotic cell lines
☒	☐	Palaeontology and archaeology
☒	☐	Animals and other organisms
☐	☒	Human research participants
☒	☐	Clinical data
☒	☐	Dual use research of concern

Methods

n/a	Involved in the study	
☒	☐	ChIP-seq
☒	☐	Flow cytometry
☒	☐	MRI-based neuroimaging

Human research participants

Policy information about studies involving human research participants

Population characteristics	Twenty healthy participants (36.1±9.6 years old; 9 females).
Recruitment	Participants were recruited through referrals from the NIH Volunteer Office, the Patient Recruitment and Public Liaison (PRPL) Office, and through ResearchMatch.org and by word of mouth and through IRB-approved advertisements. These included: 1) Flyers posted on NIH campus, campuses of universities and colleges, public places and public transportation services in the greater Washington DC/Baltimore metro areas; 2) Advertisements in electronic and printed local media (newsletters, websites, newspapers, craigslist.org, NIH and other local email distribution lists), following IRB approval. There was no direct solicitation of employees by supervisors or coworkers.
Ethics oversight	The study was approved by the IRB at the National Institutes of Health (Combined Neurosciences White Panel).

Note that full information on the approval of the study protocol must also be provided in the manuscript.
